# MicroRNA203a suppresses glioma tumorigenesis through an ATM-dependent interferon response pathway

**DOI:** 10.18632/oncotarget.22945

**Published:** 2017-12-06

**Authors:** Chuan He Yang, Yinan Wang, Michelle Sims, Chun Cai, Ping He, Hans Häcker, Junming Yue, Jinjun Cheng, Frederick A. Boop, Lawrence M. Pfeffer

**Affiliations:** ^1^ Department of Pathology and Laboratory Medicine, University of Tennessee Health Science Center, Memphis, Tennessee, USA; ^2^ Center for Cancer Research, University of Tennessee Health Science Center, Memphis, Tennessee, USA; ^3^ Department of Neurosurgery, University of Tennessee Health Science Center, Memphis, Tennessee, USA; ^4^ Department of Nephrology, Shengjing Hospital of China Medical University, Shenyang, China; ^5^ Department of Infectious Diseases, St. Jude Children's Research Hospital, Memphis, Tennessee, USA

**Keywords:** glioblastoma, ATM, miR-203, interferon, interferon signaling

## Abstract

Glioblastoma (GBM) is a deadly and incurable brain tumor. Although microRNAs (miRNAs) play critical roles in regulating the cancer cell phenotype, the underlying mechanisms of how they regulate tumorigenesis are incompletely understood. We previously showed that miR-203a is expressed at relatively low levels in GBM patients, and ectopic miR-203a expression in GBM cell lines inhibited cell proliferation and migration, increased sensitivity to apoptosis induced by interferon (IFN) or temozolomide *in vitro*, and inhibited GBM tumorigenesis *in vivo*. Here we show that ectopic expression of miR-203a in GBM cell lines promotes the IFN response pathway as evidenced by increased IFN production and IFN-stimulated gene (ISG) expression, and high basal tyrosine phosphorylation of multiple STAT proteins. Importantly, we identified that miR-203a directly suppressed the protein levels of ataxia-telangiectasia mutated (ATM) kinase that negatively regulates IFN production. We found that high ATM expression in GBM correlates with poor patient survival and that ATM expression is inversely correlated with miR-203a expression. Knockout of ATM expression and inhibition of ATM function in GBM cell lines inhibited cell proliferation and migration, increased sensitivity to apoptosis induced by therapeutic agents *in vitro*, and markedly suppressed GBM tumor growth and promoted animal survival. In contrast, restoring ATM levels in GBM cells ectopically expressing miR-203a increased tumorigenicity and decreased animal survival. Our study suggests that low miR-203a expression in GBM suppresses the interferon response through an ATM-dependent pathway.

## INTRODUCTION

Brain tumors represent an important cause of cancer-related morbidity and mortality in the United States, with malignant gliomas being among the most aggressive and difficult tumors to treat [1]. The median survival for patients with glioblastoma (GBM), the most aggressive histological subtype of glioma in adults, is only ~15 months [1]. Despite advances in our understanding of glioma development and progression, disease course and outcome for GBM patients has not significantly improved for decades [2]. Thus, treatment of GBM patients is a significant clinical challenge requiring molecular insights into tumorigenesis and novel therapeutic approaches.

MicroRNAs (miRNAs) are endogenous, small (20 to 24 nucleotide) single-stranded RNAs that regulate many fundamental biological processes [3, 4]. Although miRNAs do not encode protein, they control cellular protein expression by binding to the 3' untranslated region (UTR) of target mRNAs, promoting their cleavage or blocking their translation [3, 5]. MiRNAs play key roles in cancer initiation, progression and metastasis [4, 6], and regulate the sensitivity of tumors to radiation and chemotherapy [7]. While oncogenic miRNAs have been well studied, tumor suppressor miRNAs have been less characterized. We recently identified that miR-203a is expressed at extremely low levels in glioma patient samples and has tumor suppressive activity in glioma [8].

Interferons (IFNs) are endogenous antiviral proteins which also have anticancer activity *in vitro*, and they have been used clinically to treat various human cancers including glioma [9–11]. IFNs inhibit cell proliferation [12], and regulate the cellular responses to inducers of apoptosis [13]. Most importantly, defects in the IFN system have been linked to increased cancer susceptibility through incompletely understood mechanisms [14]. The three IFN types [Type I (α/β), II (γ) and III (λ)] bind to distinct cell surface receptors and elicit their biological effects by altering gene expression through activating JAK tyrosine kinases and STAT proteins via their tyrosine phosphorylation. Activated STATs then bind to the promoters of IFN-stimulated genes (ISGs) to directly induce ISG transcription [15–17]. ISGs regulate therapeutic resistance in preclinical cancer models [18, 19].

In this study, we examined the role of ATM, the gene mutated in ataxia telangiectasia, in the tumor suppressive action of miR-203a in GBM. We demonstrate that ATM is a direct miR-203a target in GBM, and miR-203a suppresses ATM expression. ATM is a central regulator of genotoxic and metabolic stress responses [20], and negatively regulates IFN production [21]. By suppressing ATM levels, we show that miR-203a promotes the IFN response pathway as evidenced by induction of IFN production, ISG expression, and high basal tyrosine STAT phosphorylation. We show by genetic and pharmacological approaches that ATM promotes GBM tumorigenicity *in vitro* and *in vivo*, and decreases the sensitivity of GBM to therapeutic agents. Our results indicate that ATM might be an important therapeutic target for the treatment of GBM.

## RESULTS

### MiR-203a promotes the IFN response pathway in GBM cells

**Figure 1:**
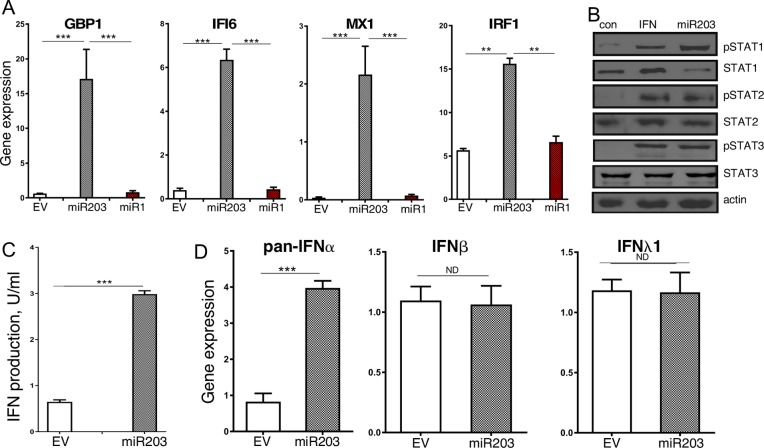
Enforced miR-203a expression results in constitutive activation of the IFN response pathway MT330 GBM cells were transduced with miR-203a-encoding, miR1-encoding, or empty-vector (EV) lentivirus. **(A)** RNA extracts were assayed for ISG expression relative to actin expression. **(B)** Protein lysates were immunoblotted for STAT and pTyr-STAT proteins as indicated. As a positive control for STAT activation lysates were also prepared from empty vector-transduced cells treated with IFNα (1000 IU/ml for 30 min). **(C)** Media from cells was assayed on human CaKi cells expressing an ISRE-driven luciferase reporter construct, and results expressed as IFN IU/ml. **(D)** RNA extracts were prepared, and qPCR was performed using a pan-IFNα primer, IFNβ and IFNλ primers, and gene expression normalized to actin expression.

We previously demonstrated that miR-203a is expressed at low levels in GBM patients, and ectopic miR-203a expression in GBM cell lines inhibited GBM tumorigenesis *in vitro* and *in vivo* [8]. To identify potential miR-203a target genes we performed microarray analysis on MT330 and SJG2 GBM cells with enforced miR-203a expression, and found the spontaneous induction of IFN-stimulated genes (ISGs), including IFI6, IFT20, IRF1, ISG15, ISG20, and MX1 (Supplementary Table 1), which was surprising as miRNAs typically downregulate gene expression and these ISGs did not have identifiable miR-203a binding sites in their 3'UTRs. To determine whether the effect on ISG expression was selective for miR-203a, GBM cells with enforced miR1 expression (another miRNA expressed at relatively low levels in GBM cells) were also isolated, and gene expression in RNA extracts was determined by qPCR. Enforced miR-203a resulted in high IFI6, MX1, GBP1 and IRF1 expression levels, while enforced miR1 had no effect on the expression of these genes (Figure 1A). Since IFN-induced tyrosine phosphorylation of STAT proteins regulates ISG expression, lysates were prepared from miR-203a-enforced cells and immunoblotted for tyrosine phosphorylated STAT proteins. Cells with enforced miR-203a showed high basal tyrosine phosphorylation ofSTAT1, STAT2 and STAT3, roughly equivalent to that induced by short-term IFN treatment (Figure 1B). While basal levels of STAT2 and STAT3 were unaffected by miR-203a expression, enforced miR-203a selectively suppressed STAT1 levels consistent with our previous findings [8]. Since STAT2 is activated by type I IFN (IFNα/β) but not by type 2 IFN (IFNγ), these data indicated that miR-203a activated the IFNα/β signaling pathway. Furthermore, using a reporter assay driven by an ISG promoter, we found significantly higher IFN levels in conditioned media from miR-203a enforced MT330 cells as compared to control MT330 cells (Figure 1C). Moreover, using IFN subtype-specific PCR primers, we found that miR-203a-enforced MT330 and SJG2 GBM cells have significantly higher IFNα gene expression than control cells but no difference in IFNβ or IFNλ gene expression (Figure 1D). Taken together our results show that miR-203a promotes the induction of the IFNα signaling pathway.

### ATM is a miR-203a target gene

**Figure 2:**
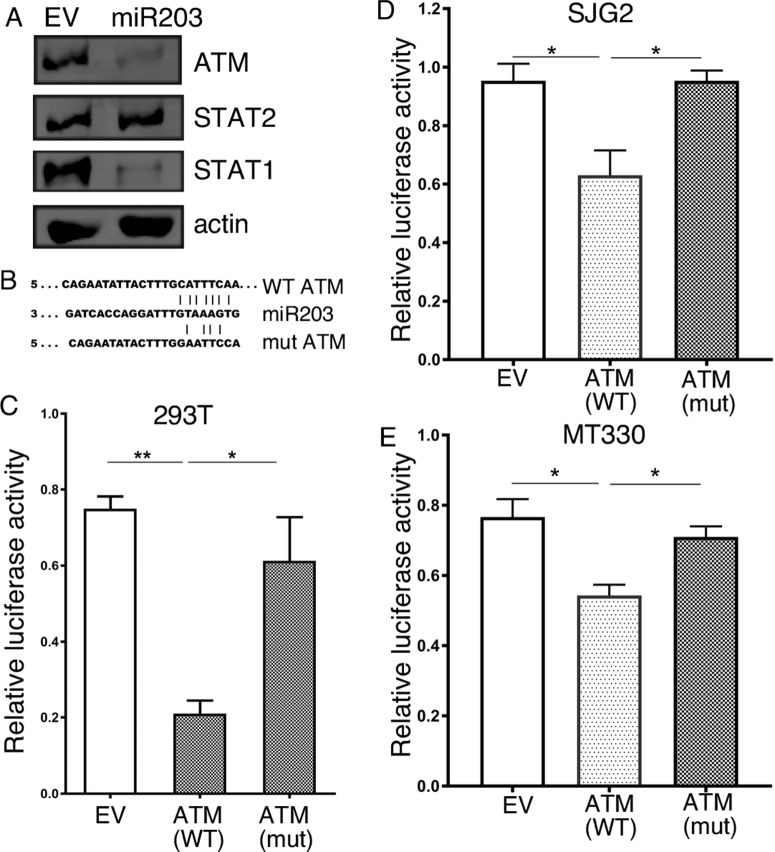
ATM is a miR-203a target gene **(A)** Protein lysates were prepared from MT330 GBM cells that were transduced with miR-203a-encoding or empty-vector (EV) lentivirus, and immunoblotted as indicated. **(B)** Sequence alignment of the miR-203a binding sequence with the 3'UTR of the wild-type (WT) and mutant (mut) ATM constructs used in the following reporter assays. 293T **(C)**, SJG2 **(D)** and MT330 **(E)** cells were transiently cotransfected with miR-203a plasmid, pSV40-Renilla, and with plasmid empty-vector (pcDNA3.1-Luc), WT (pcDNA3.1-Luc-wtUTR) or mutant (pcDNA3.1-Luc-muUTR) ATM reporter plasmids. The ratio of luciferase and Renilla activities was determined at 24 hr post-transfection.

Since miR-203a promotes the induction of the IFNα signaling pathway, we next examined whether miR203a targeted genes in the IFN response. By binding to target mRNAs and silencing their expression, miRNAs control cellular protein expression. By bioinformatics analysis, we identified ATM as a potential miR-203a target, which has been shown to suppress the IFN response [21]. Consistent with ATM being a miR-203a target, immunoblotting of whole cell lysates prepared from empty vector (EV) and miR-203a-transduced MT330 cells showed that protein levels of ATM and STAT1 [known miR-203a target [8]] were markedly lower in MT330 cells with enforced miR-203a expression, but the levels of STAT2 and actin were unaffected (Figure 2A). Using the miR-203a core seed sequence (GTAAAGT), we identified a complementary binding site in the 3' UTR of ATM (Figure 2B). To determine whether ATM was a direct miR-203a target, the 3'UTR of ATM mRNA containing the predicted miR-203a target sequence as well as a corresponding mutated sequence were linked to luciferase, and a dual-luciferase (pcDNA3.1-Luc) reporter system was employed to evaluate miRNA:mRNA interactions [8, 22, 23]. Overexpression of miR-203a in HEK293T cells downregulated luciferase activity of the ATM-driven wild-type reporter construct, while a construct driven by the mutated miR-203a binding sequence was unaffected by miR-203a overexpression (Figure 2C). Reporter assays performed in MT330 and SJG2 GBM cell lines showed qualitatively similar results, i.e. overexpression of miR-203a resulted in decreased luciferase activity of the reporter construct driven by wild-type ATM sequence, but not on the construct driven by the mutant ATM sequence (Figure 2D, 2E). Taken together, these results show that ATM is a bona fide miR-203a target gene.

### High ATM expression correlates with poor patient survival

**Figure 3:**
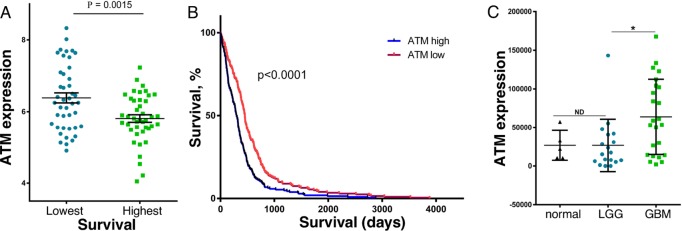
ATM expression in brain cancer patient samples, and the relationship to patient survival **(A)** GBM patients in the TCGA database with the lowest (43 patients) and highest survival (42 patients) were compared for ATM expression. **(B)** Kaplan-Meier analysis of the survival of GBM patients (422 patients) in the TCGA database that were grouped into high (213 patients) and low (209 patients) ATM expression. **(C)** RNA was extracted from age-matched and sex-matched FFPE patient biopsies identified as GBM (24 samples), LGG (18 samples) or normal brain tissue (5 samples), and ATM expression was determined by qPCR (n = 3), and normalized to actin expression [37].

Since miR-203a is a tumor suppressor in GBM we hypothesized that ATM has pro-tumorigenic activity in GBM. To characterize the potential role of ATM in GBM, we examined the relationship between ATM expression and patient survival from the TCGA dataset. Since GBM patient survival remains poor with a median overall survival of ~ 14 months, we examined ATM expression in GBM patients with the top 10% and lowest 10% survival. As shown in Figure 3A, we found that GBM patients with the lowest overall survival have significantly higher ATM expression as compared to patients with the best overall survival (p=0.0015). As an alternative approach, we examined the relationship between ATM expression and individual patient survival in the TCGA dataset. As shown in Figure 3B, ATM-high GBM patients had significantly shorter survival than ATM-low GBM patients (p<0.0001). In addition, ATM expression was determined by qPCR in RNA isolated from FFPE biopsy specimens from primary low-grade glioma (WHO classification I and II), GBM (WHO classification IV), and histologically benign brain samples. ATM expression was highest in GBM, and markedly lower in both benign brain tissue and low-grade glioma (Figure 3C). Taken together, these results show that high ATM expression in glioma is associated with poor patient survival.

### Knockout of ATM in GBM cells impairs NFκB activation, cell proliferation and migration, and sensitizes cells to apoptosis

**Figure 4:**
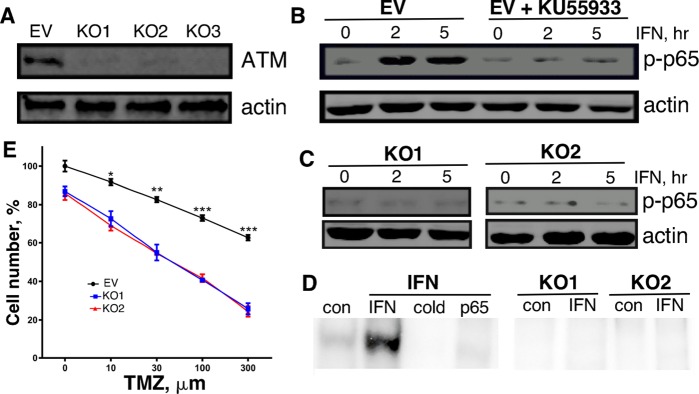
Characterization of ATM-KO MT330 cells **(A)** Cell lysates were prepared from pools of ATM-KO MT330 cells using different gRNAs and immunoblotted as indicated. **(B, C)** Lysates were prepared from the following cells treated with IFN (1000 IU/ml): EV-transduced cells in the presence or absence of the (B) ATM inhibitor (10 μM KU55933, 1 hr pretreatment), (C) ATM-KO1 or ATM-KO2 cells, and immunoblotted with an antibody specific for phosphorylated p65. **(D)** Gel shift assays were performed on nuclear lysates of control and IFN-treated (1000 U/ml, 30 min) EV, ATM-KO1, or ATM-KO2 MT330 cells using an ^32^P-labeled oligonucleotide NFκB probe as performed previously [38]. A 50-fold excess of unlabeled probe (cold) was used to show binding specificity, and the presence of p65 was shown by supershift with anti-p65 antibody (p65).

Since ATM was expressed at relatively high levels in GBM patient samples and cell lines, we examined the biological consequences of ablating ATM expression in GBM cells by CRISPR/Cas9 mediated knockout (KO). As determined by immunoblotting of cell lysates, ATM expression was nearly completely ablated in ATM-KO1, ATM-KO2 and ATM-KO3 MT330 cells (Figure 4A). Since ATM is upstream of NFκB activation in the signaling pathway of various cytokines including IFN [21], we examined the effect of the ATM inhibitor (KU55933) and ATM-KO on IFN-induced NFκB activation, using the serine phosphorylation of the p65 subunit of NFκB as a measure of activation [24]. While both IFN and TNF induced serine phosphorylation of p65 in MT330 cells, KU55933 pretreatment blocked p65 serine phosphorylation (Figure 4B and Supplementary Figure 1). In addition, IFN did not induce p65 phosphorylation in ATM-KO1 or ATM-KO2 MT330 and SJG2 cells (Figure 4C and Supplementary Figure 2). To further determine the effect of ATM-KO on NFκB activation, nuclear extracts were subjected to gel shift assays with an NFκB specific oligonucleotide probe. IFN induced NFκB-dependent DNA binding, which was super-shifted with anti-p65 antibody and was competed by a 50-fold excess of cold probe. (Figure 4D). In contrast, no detectable NFκB activation was induced by IFN in either ATM-KO1 or ATM-KO2 MT330 cells, providing additional evidence that ATM promotes NFκB activation. Taken together, our results show that knockout of ATM in GBM cells blocks NFκB activation, which is downstream of ATM signaling.

**Figure 5:**
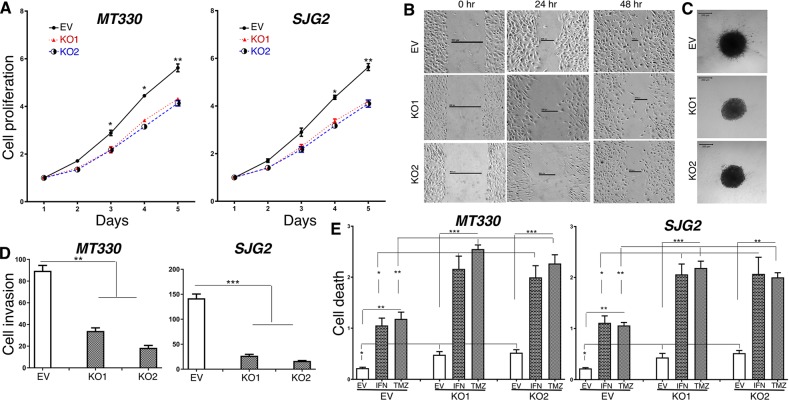
The effects of ATM-KO on glioma cell proliferation, invasion and sensitivity to induction of apoptosis Cell proliferation **(A)**, wound healing **(B)**, 3D cell invasion **(C)**, transwell invasion **(D)** ofEV and ATM-KO GBM cells. **(E)** EV and ATM-KO GBM cells were treated for 24 hr with IFN (1,000 IU/ml) or TMZ (100 μM) and analyzed for apoptosis by cell death detection ELISA assays.

We next examined the biological consequences of ATM-KO in GBM cells *in vitro*. The effect of ATM-KO on cell proliferation was monitored daily by cell counting, and ATM-KO was found to inhibit the proliferation of both MT330 and SJG2 glioma cells (Figure 5A). In addition, ATM-KO inhibited cell migration as evidenced by impaired wound healing, decreased cell invasion in 3-dimensional spheroid assays, and decreased numbers of migrating cells in matrigel-coated filter invasion assays (Figure 5B-5D). In addition, apoptosis in ATM-KO GBM cells was determined by a cell-death ELISA after treatment with IFN or temozolomide (TMZ), which induces apoptosis through DNA strand breaks [25]. While basal apoptosis was slightly increased by ATM-KO expression, apoptosis induced by IFN and TMZ was markedly increased in both MT330 and SJG2 cells with ATM-KO (Figure 5E). Similar results were obtained when apoptotic cells were enumerated by flow cytometry of annexin V-stained cells (Supplementary Figure 3). Moreover, treatment with the ATM inhibitor KU55933 also induced GBM cell apoptosis (Supplementary Figure 4). Taken together, these results show that ATM-KO inhibits GBM cell proliferation and cell migration, and enhances sensitivity of GBM cells to inducers of apoptosis. These biological consequences of ATM-KO closely mirror the effect of enforced miR-203a expression in GBM cells [8].

### Enforced miR-203a expression and ATM knockdown inhibits GBM tumorigenesis

**Figure 6:**
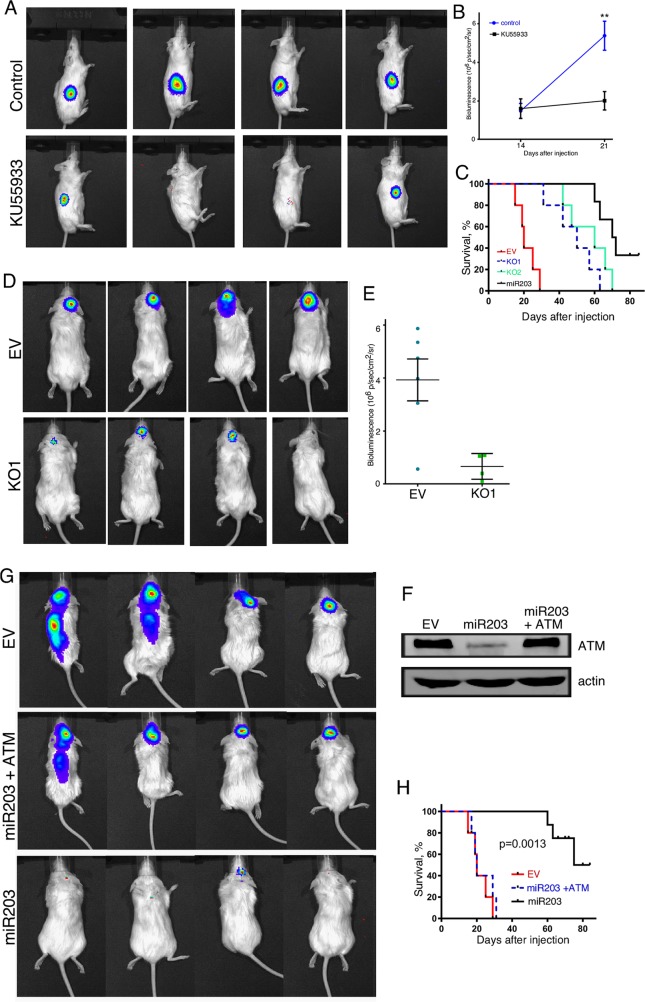
The effects of ATM-KO and KU55933 treatment on tumor formation by GBM cell lines **(A)** Live animal imaging of NSG mice that were injected subcutaneously with 10^6^ MT330 cells, and after 14 days were treated with the KU55933 ATM inhibitor (10 mg/kg mouse by IP injection, thrice per week) or vehicle (6 mice per group) and representative images are shown at 21 days post-injection. **(B)** Quantitation of bioluminescence of mice in each group at 14 and 21 days after injection of GBM cells. **(C)** Kaplan-Meier analysis of survival data of the mice injected in Panel A was performed. **(D)** Live animal imaging of NSG mice (6 mice/group) at 6 weeks after intracranial injection with 10^6^ EV or ATM-KO MT330 cells, and **(E)** bioluminescence signal quantified. No bioluminescent signal was detected in mice injected with ATM-KO2 MT330 cells. **(F)** Cell lysates were prepared from EV transduced MT330 cells (EV), miR-203a enforced MT330 cells (miR-203), or miR-203a enforced MT330 cells that had ATM expression restored (miR-203+ATM) and immunoblotted for ATM or actin. **(G)** NSG mice (5 mice/group) were injected intracranially with 10^6^ EV MT330 cells, miR-203a enforced cells or cells that had ATM expression restored, and tumor growth was determined by live animal imaging at 6 weeks, and **(H)** Kaplan-Meier analysis of survival data was performed.

We then determined the effect of inhibiting ATM function *in vivo* on the tumorigenicity of GBM cells by pharmacologically inhibiting ATM or by knocking it out utilizing a genetic approach. NSG mice were injected subcutaneously with MT330 GBM cells and after 2 weeks of tumor formation mice were treated with the pharmacological ATM inhibitor KU55933. Tumor growth was determined by live animal imaging. After only one week, inhibiting ATM activity by KU55933 treatment markedly suppressed tumor growth of MT330 GBM cells (Figure 6A and 6B). In addition, the role of ATM was also determined in the orthotopic microenvironment for GBM by intracranial injections of luciferase-expressing control and ATM-KO MT330 cells, and tumorigenesis followed by live animal imaging after D-luciferin injection. Significant bioluminescent signal was evident throughout the brain of mice injected with control MT330 cells demonstrating marked tumor induction and invasion. In contrast, brain tumor formation was significantly reduced in mice injected with ATM-KO1 or ATM-KO2 cells (Figure 6D and 6E, and data not shown). Moreover, as shown in Figure 6C, survival was significantly prolonged in mice injected intracranially with ATM-KO cell lines. Moreover, while mice injected with MT330 cells showed early marked neurological deficits, such as difficulty in movement and feeding, in mice injected with ATM-KO cells these deficits were delayed for several weeks (data not shown). The inhibitory effect on tumor formation and increase in animal survival upon ATM-KO and/or pharmacologic inhibition of ATM function mirrored what we observed upon enforced miR-203a expression and was consistent with our previous findings [8]. In cells ectopically-expressing miR-203a, we restored ATM expression by an ORF construct that lacks the miR-203a binding site (Figure 6F), and examined the effect on cell tumorigenicity upon intracranial injection. Although enforced miR-203a expression in MT330 cells markedly reduced tumorigenicity, restoration of ATM expression increased tumorigenicity, similar to that observed with control MT330 cells (Figure 6G). Furthermore, animal survival was markedly reduced after restoring ATM expression (Figure 6H). Taken together, these results show that ATM has pro-tumorigenic action on GBM *in vivo*.

## DISCUSSION

Since GBM is a malignancy with an extremely poor prognosis, it is critical to identify new molecular pathways that can be targeted for treatment. In the present study, we show that ectopic expression of miR-203a in low miR-203a-expressing GBM cell lines promotes the interferon (IFN) response pathway by several approaches. First, we showed that enforced miR-203a expression in GBM cells lines resulted in high expression of several “classical” ISGs (IFI6, MX1, GBP and IRF1) by microarray and qPCR analysis. Then we found in GBM cells with enforced miR-203a expression constitutively high levels of activated STAT1, STAT2 and STAT3 as evidenced by their tyrosine phosphorylation. In addition, we also found increased levels of IFN in culture supernatants of miR-203a-expressing GBM cells, and increased IFNα gene expression levels. Taken together our results suggest that enforced miR-203 expression in GBM cells promotes the selective induction of IFNα gene expression resulting in STAT activation and high ISG expression.

Interferons (IFNs) have anticancer activity *in vitro*, and have been used clinically to treat various human cancers including brain cancer [9–11]. Defects in the IFN response pathway increase cancer susceptibility through incompletely understood mechanisms [14]. Moreover, radiation therapy and chemotherapy induce ISG expression, correlating with antitumor response [26–28]. These results indicate that the IFN response pathway may play a key role in regulating tumorigenesis. To the best of our knowledge, our studies are the first to link miR-203a specifically, or any miRNA in general, to regulating the IFN response pathway in any form of cancer. Our finding that miR-203a promotes the selective expression of IFNα and not IFNβ is somewhat surprising, since IFNα and IFNβ genes are usually co-regulated upon their induction. This may reflect the different locations of the genes for the multiple IFNα subtypes and the single IFNβ on human chromosome 9. While all IFNα genes are clustered together in the centromeric region of chromosome 9, the IFNβ gene is located at the extreme telomeric end of the chromosome [29].

Since miRNAs usually suppress the expression of their target genes, we focused on miR-203a-downregulated genes that could potentially inhibit the IFN response. By this approach, we identified ATM as a potential miR-203a target, and found that enforced miR-203a expression inhibited the protein levels of ATM. In addition, using luciferase reporter assays driven by wild-type and mutant 3'UTR sequences of ATM, we showed that ATM was directly regulated by miR-203a. These results identified ATM as a *bona fide* miR-203a target in GBM cells, which is consistent with the finding that miR-203 binds to the 3'UTR of ATM and suppresses ATM levels in colorectal cancer cells [30]. In addition, we found that ectopic miR-203a expression resulted in IFN production, ISG expression and STAT activation, which is consistent with our finding that inhibiting ATM induced IFN production in GBM cells (Supplementary Figure 5).

We previously found that miR-203a has tumor suppressive function in GBM by suppressing genes that promote tumorigenesis [8]. We then examined the relationship in GBM patient tumor samples between ATM expression and survival or severity of disease. We found that GBM patients with the lowest overall survival had significantly higher levels of ATM expression, and that high-ATM expressing glioma patients had significantly shorter overall survival as compared to low-ATM expressing glioma patients. These results are consistent with our hypothesis that ATM promotes GBM tumorigenesis.

To characterize the functional role of ATM in GBM cells we ablated ATM expression by CRISPR/Cas9-mediated knockout. Consistent with its role in regulating NFκB activity [21], ATM-KO in GBM cells markedly inhibited NFκB activation as evidenced by p65 serine phosphorylation and gel shift assays with a NFκB probe. Similarly, NFκB activation was blocked when ATM function was pharmacologically inhibited with KU55933. Most importantly, we demonstrated that knockout of ATM expression and inhibition of ATM function in GBM cell lines inhibited cell proliferation and migration, increased sensitivity to apoptosis induced by agents that have been used to treat GBM patients in the clinic, mirroring the effects of enforced miR-203a expression [8].

In addition, while genetic and pharmacologic inhibition of ATM markedly suppressed GBM tumor growth and promoted animal survival, restoring ATM levels in GBM cells ectopically expressing miR-203a increased tumorigenicity and decreased animal survival. Taken together these results indicate that ATM plays a pro-tumorigenic function in GBM. The pro-tumorigenic role of ATM in brain cancer is somewhat unexpected, since ATM deletion accelerated tumorigenesis in an *in vivo* mouse model of human GBM [31]. However, consistent with our findings genetic inactivation of the ATM pathway was protective against GBM formation [32]. Although ATM has often been linked to cancer development [20], ATM signaling can also be advantageous to cancer cells, particularly in resistance to radiation and chemotherapy. For this reason, ATM inhibitors have been developed for use in cancer therapy.

Of particular translational importance is our finding that genetic KO of ATM or treatment with an ATM inhibitor sensitizes GBM cells to various chemotherapeutic agents, such as the alkylating agent TMZ and the cytokine IFN. ATM plays a critical role in the cellular response to double-strand breaks and other genotoxic agents [20]. Therefore, our results showing that ATM ablation or functional inhibition sensitizes GBM cells to various chemotherapeutic agents are consistent with the critical role that ATM plays in the DNA damage response. Furthermore, ATM inhibition has been shown to sensitize GBM cell lines to radiation-induced damage in a p53-mutant dependent manner [33]. However, both GBM cell lines employed in our studies express wild-type p53, indicating that ATM may function in both a p53-dependent and p53-independent manner. In future, studies it will be important to define the molecular pathway through which ATM functions in GBM, as well the cross-talk between ATM and the NFκB pathway [34].

We report that high ATM expression in GBM correlates with poor patient survival and that ATM expression is inversely correlated with miR-203a expression, strongly suggesting ATM may play an important role in GBM tumorigenesis and has potential as a therapeutic target to sensitize GBM to chemotherapy and/or radiotherapy. Thus, we believe that ATM expression is relatively high in GBM in part due to low miR-203a expression, rendering GBM relatively resistant to therapy. Thus, within the context of DNA repair ATM functions as a tumor suppressor (classical function). However, within the context of pathways such as in the suppression of IFN induction ATM may promote tumorigenesis. In summary, our findings suggest that low miRNA203a expression in GBM suppresses the IFN response by an ATM-dependent pathway, and that miR-203a has tumor suppressive function in GBM by inhibiting the pro-tumorigenic function of ATM.

## MATERIALS AND METHODS

### Biological reagents and cell cultures

The biological activity of recombinant human interferon (IFNcon1, InterMune) is expressed in terms of international reference units/ml using the human NIH reference standard [35]. Antibodies against the following proteins were used: STAT1, STAT2, p-STAT1, p-STAT2, phospho-p65 and actin (Santa Cruz Biotechnology); STAT3 (BD Transduction Laboratories); pTyr-STAT3, (Abcam) and ATM (Cell Signaling). MT330 (UTHSC Department of Neurosurgery) and SJG2 (St. Jude Children's Research Hospital) GBM cell lines were grown in DMEM containing 10% fetal bovine serum (Atlanta Biologics) supplemented with penicillin (100 IU/ml) and streptomycin (100 μg/ml) at 37°C with 5% CO_2_.

### Gene expression analysis

Total RNA was isolated using RNeasy Mini kit (Qiagen) from MT330 and SJG2 cells, and submitted to the UTHSC Center of Genomics and Bioinformatics (Memphis, TN) for hybridization to Human-HT12 BeadChips (Illumina). Microarray data analysis was carried out as described previously [36]. Gene expression was determined by quantitative real time PCR (qPCR) using the following gene-specific primers [37]: pan-IFNα, 5'- CACACAGGCTTCCAGGCATTC-3' (forward) and 5'- TCTTCAGCACAAAGGACTCATCTG-3' (reverse); IFNβ, 5'-TGCTCTCCTGTTGTGCTTCTCCAC -3' (forward) and 5'- ATAGATGGTC AATGCGGCGTCC-3' (reverse); IFNλ, 5'- CGCCTTGGAAGAGTCACTCA-3' (forward) and 5'- GAAGCCTCAGGTCCCAATTC-3' (reverse); IF16, 5'- GGTCTGCGATCCTGAATGGG -3' (forward) and 5'-TCACTATCGAGATACTTGTGGGT -3' (reverse); MX1, 5'- TCCCACCCTCTATTACTGAATGG-3' (forward) and 5'- GGGAAGGGCAACTCCTGAC-3' (reverse); GBP1, 5'- AGGAGTTCCTTCAAAGATGTGGA-3' (forward) and 5'- TTCTGAACAAAGAGACGATAGCC-3' (reverse); IRF1, 5'- ATGCCCATCACTCGGATGC-3' (forward) and 5'- CCCTGCTTTGTATCGGCCTG-3' (reverse). For miRNA expression, total RNA (5 μg) was reverse-transcribed into first-strand cDNA and 30 ng of cDNA was used as a template for the PCR reaction with a forward primer specific to the mature miR-203a sequence, and the following mature miR-203a sequence (5'- GTGAAATGTTTAGGACCACTAG-3'). SYBR Green-based real-time PCR was performed and miRNA or mRNA expression normalized relative to U6 or β-actin expression, respectively. In addition, gene expression was determined in RNA isolated from de-identified formalin-fixed paraffin-embedded (FFPE) patient biopsy specimens (UTHSC Tissue Services Core) as previously described [22].

### Lentiviral-mediated miR-203a and ATM expression

Stable pools of miR-203a lentiviral transduced overexpressing GBM cells were isolated as previously described [37]. Restoration of ATM levels was performed by lentiviral transduction with lentivirus expression vector encoding the ATM open reading frame (EX-Y1898-Lv122 from GeneCopeia).

### Immunoblot analysis

Total cell lysates (25 μg) were separated by SDS-PAGE, immunoblotted with the indicated antibodies and visualized as previously described [22].

### Construction of luciferase reporter gene plasmids and reporter assays

The 3' UTR of ATM containing the predicted miR-203 a binding site was amplified by PCR from genomic DNA of human 293T cells. After digestion with XhoI and BamHI, the PCR product was purified and cloned into pcDNA3.1-luc, resulting in the wild-type ATM reporter plasmid, pcDNA3.1-Luc-wtUTR. The mutant ATM reporter plasmid pcDNA3.1-Luc-muUTR was constructed by mutating the miR-203a binding site in the 3'UTR of ATM using PCR based site-directed mutagenesis (Stratagene). The primers for amplifying the wild-type 3'-UTR were 5'- GATACTCGAGCTCGTGTATTAGTGAGTATAATCTC-3' and 5'- GCGGATCCGCAAGGCTAAAGAGTAGATTAA-3', and the primers for mutant construct were 5'- GAATTCGAATTACAACTTACCTTGGTGTATCT-3' and 5'- CAAAGTAATATTCTGAACAGTTCTCCAGAAGT-3'. Reporter gene binding assays were performed as previously described [22] by co-transfecting cells using wild-type and mutant reporter plasmids pcDNA3.1-Luc-wtUTR and pcDNA3.1-Luc-muUTR with miR-203a overexpressing plasmid, respectively.

### IFN assays

IFN bioactivity was determined using human CaKi cells stably transduced with a lentivirus containing a luciferase reporter gene driven by five tandem ISREs. Reporter cells were exposed to IFN-containing cell supernatants, and luciferase activity determined after ~24-hr incubation. Standard curves with human IFNα were used to calculate IFN supernatant levels.

### Gel shift assays

Nuclear extracts (10 μg) were incubated with ^32^P-labeled κB probe (5'-AGTTGA-GGGGACTTTCCCAGG-3') derived from an NFκB binding sequence in the immunoglobulin gene promoter, and subjected to gel shift assays [38]. To define the presence of p65 in DNA-protein complexes, nuclear extracts were preincubated with a 1:50 dilution of anti-p65 antibodies at 25°C for 20 min before the assay.

### Tumor formation in mice

Animal experiments were performed in accordance with a protocol approved by the Institutional Animal Care and Use Committee of the University of Tennessee Health Science Center. Xenografts were established in five-week-old male NOD.Cg-Prkdc^scid^ Il2rg^tm1Wjl^/SzJ (NSG) mice (Jackson Laboratory) by injection of GBM cells (1×10^6^) directly into the flanks [39]. Tumors were measured weekly with a handheld caliper. In addition, luciferase-expressing cells (10^6^) were injected stereotactically into the superficial brain parenchyma of NSG mice through a burr hole in the skull as previously described [40]. NSG mice were injected with D-luciferin and subjected to live animal imaging weekly to quantify bioluminescence [40, 41].

### TCGA data query

We queried the TCGA portal for all GBM samples with Level 3 miRNA or mRNA expression data. The dataset was filtered for samples having expression data for miR-203, ATM and accompanying clinical data, yielding a final set of 422 GBM samples. Statistical analyses were performed using Graphpad Prism.

### Cell proliferative, migration, invasion, wound healing and apoptosis assays

For cell proliferation analysis, cultures were plated and enumerated in a Coulter Counter as previously described [42]. Matrigel-coated filter invasion assays using transwell inserts (BD Biosciences) were performed as previously described [43]. Cultrex 96-well 3D spheroid cell invasion assays (Millipore Sigma) were performed according to the manufacturer's protocol, and images were taken on a Nikon LSM700 confocal microscope at 4 days after seeding. Wounds were created in confluent cell monolayers with a sterile 1000 μL pipette tip, and phase-contrast images were recorded to assess wound healing. Apoptosis was monitored by cell death ELISA assays as previously described [8].

### Generation of ATM-KO cells

The lentiviral CRISPR/Cas9 mediated ATM knockout vectors were constructed by cloning three ATM gRNAs (gRNA1: 5'-CCAAGGCTATTCAGTGTGCG-3'; gRNA2: 5'-TGATAGAGCTACAGAACGAA-3'; and gRNA3: 5'- CCTCGCACACTGAATAGCCT-3') into the BsmII site of lentiviral vector pLenti CRISPR V2. A control vector was constructed by inserting the EGFP gRNA sequence into the lentiviral vector. Lentivirus were produced by packaging in 293FT cells as we published previously [44]. Stable pools of ATM-KO cells were generated by transducing GBM cells with the lentiviral CRIPSR/Cas9 vectors and selected with 5 μg/ml puromycin.

### Statistical analyses

At least two independent experiments were performed in duplicate, and data are presented as means ± sd. ANOVA and post-hoc least significant difference analysis or Student's t-tests were performed. p values < 0.05 (^*^), 0.01 (^**^) and 0.001 (^***^) were considered statistically significant.

## SUPPLEMENTARY MATERIALS FIGURES AND TABLES




