# Supplementary material for: MicroRNA203a suppresses glioma tumorigenesis through an ATM-dependent interferon response pathway
Source: Oncotarget. 2017 Dec 6;8(68):112980–91. doi: 10.18632/oncotarget.22945 (PMC5762567; doi:10.18632/oncotarget.22945)
Supplement: Supplementary file 1 [file oncotarget-08-112980-s001.pdf]

MicroRNA203a suppresses glioma tumorigenesis through an ATM-dependent interferon response pathway

SUPPLEMENTARY MATERIALS

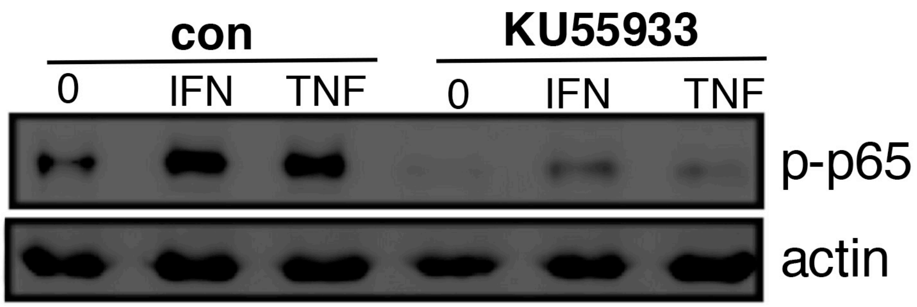

Supplementary Figure 1: KU55933 treatment blocks the induction of NFκB activity. Cell lysates were prepared from MT330 cells treated with IFN (1000 IU/ml), or TNF (20 ng/ml) in the presence or absence of the ATM inhibitor (10 μM KU55933, 1 hr pretreatment), and immunoblotted with an antibody specific for phosphorylated p65.

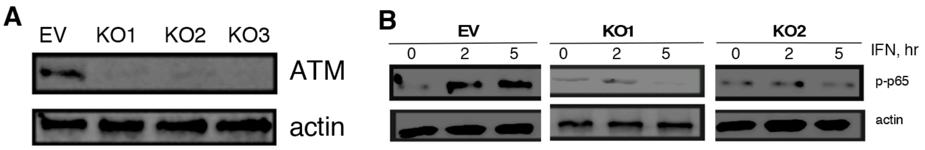

Supplementary Figure 2: Characterization of ATM-KO SJG2 cells. (A) Cell lysates were prepared from pools of ATM-KO SJG2 cells using different gRNAs and immunoblotted as indicated. **(B)** Lysates were prepared from ATM-KO1 or ATM-KO2 cells, and immunoblotted with an antibody specific for phosphorylated p65.

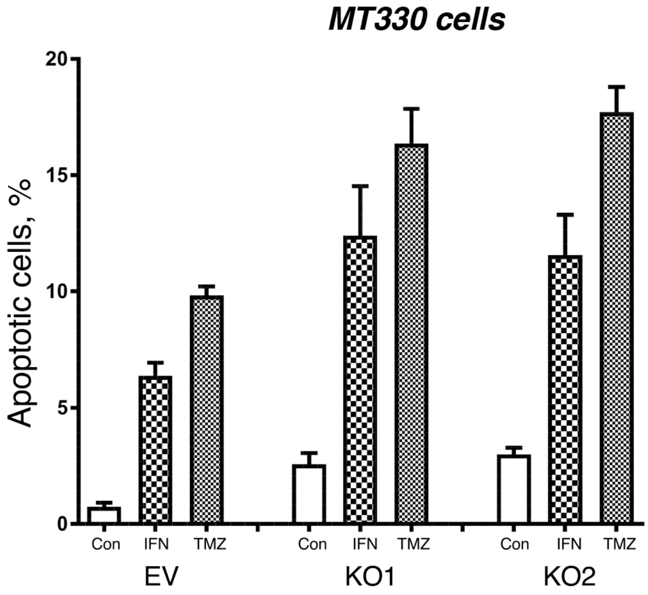

Supplementary Figure 3: The effects of ATM-KO on the induction of apoptosis. MT330 cells were treated for 24 hr with IFN (1,000 IU/ml) or TMZ (100 μM), and apoptosis was monitored by flow cytometry using the Annexin V-FITC apoptosis detection kit (BD Biosciences).

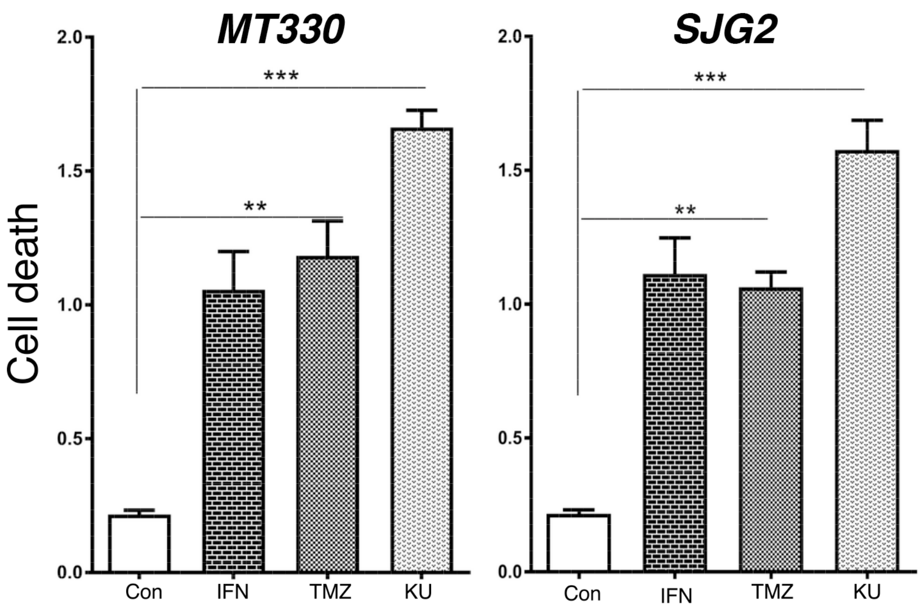

Supplementary Figure 4: The effects of ATM inhibition on the induction of apoptosis. GBM cells were treated for 24 hr with IFN (1,000 IU/ml), TMZ (100 μM) or KU55933 (10 μM) and analyzed for apoptosis by cell death detection ELISA assays.

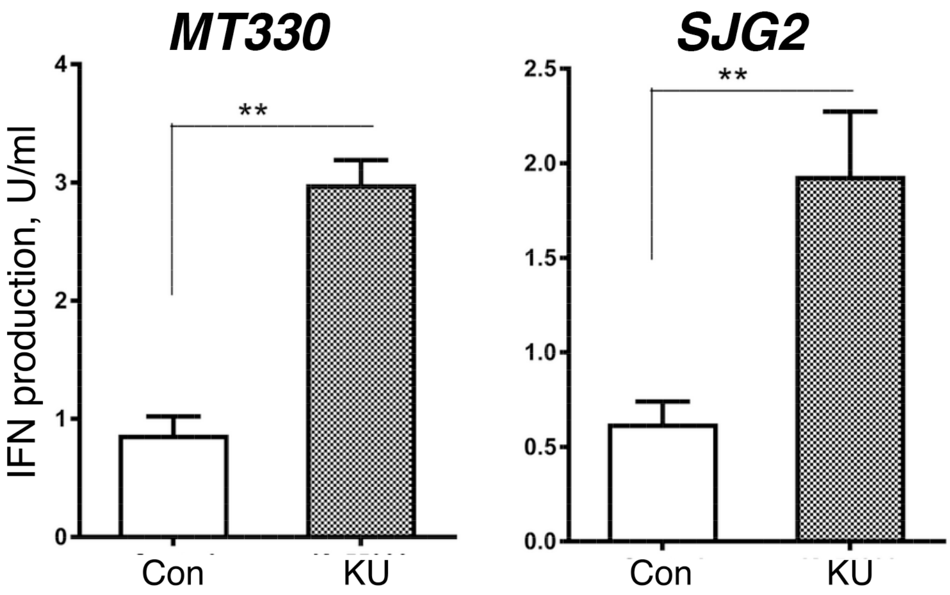

Supplementary Figure 5: The effects of ATM inhibition on the induction of IFN gene expression. Media from GBM cells treated for 24 hr with KU55933 (10 μM) was assayed on human CaKi cells expressing an ISRE-driven luciferase reporter construct, and results expressed as IFN IU/ml.

Supplementary Table 1: Genes induced by enforced miR203a expression in MT330 and SJG2 GBM cells.

See Supplementary File 1
