# Supplementary material for: MicroRNA203a suppresses glioma tumorigenesis through an ATM-dependent interferon response pathway
Source: Oncotarget. 2017 Dec 6;8(68):112980–91. doi: 10.18632/oncotarget.22945 (PMC5762567; doi:10.18632/oncotarget.22945)
Supplement: Supplementary file 2 [file oncotarget-08-112980-s002.doc]

**Supplementary Table 1: Genes induced by enforced miR203a expression**

**in MT330 and SJG2 GBM cells**

**MT330 cells** **SJG2 cells**

| Gene | EV | miR-203a | Fold change |  | Gene | EV | miR-203a | Fold change |
| --- | --- | --- | --- | --- | --- | --- | --- | --- |
| HLA-F | 516.9584 | 4314.437 | 8.345811 |  | HLA-F | 4328.384 | 6521.582 | 1.506701 |
| HMOX1 | 1344.233 | 11953.25 | 8.892246 |  | HMOX1 | 1156.266 | 6341.93 | 5.484837 |
| IER3 | 8673.147 | 14466.02 | 1.667909 |  | IER3 | 19922.35 | 43683.13 | 2.19267 |
| IFI6 | 1984.49 | 5534.992 | 2.789126 |  | IFI6 | 2255.033 | 6405.831 | 2.840682 |
| IFT20 | 240.1123 | 656.0196 | 2.732137 |  | IFT20 | 508.369 | 1820.832 | 3.581712 |
| IGFBP6 | 189.0256 | 1250.13 | 6.613549 |  | IGFBP6 | 2012.476 | 7067.295 | 3.511741 |
| IP6K1 | 508.433 | 1360.535 | 2.675938 |  | IP6K1 | 812.1444 | 1400.692 | 1.724683 |
| IPO4 | 2898.279 | 8761.004 | 3.02283 |  | IPO4 | 1039.093 | 1753.391 | 1.687425 |
| IRF1 | 219.24 | 495.3948 | 2.2596 |  | IRF1 | 186.47 | 389.126 | 2.086802 |
| ISG15 | 2628.452 | 6795.88 | 2.585506 |  | ISG15 | 999.283 | 2800.578 | 2.802587 |
| ISG20 | 182.3823 | 3682.383 | 20.19046 |  | ISG20 | 962.9981 | 3666.224 | 3.807094 |
| ISG20L2 | 830.8995 | 1251.365 | 1.506037 |  | ISG20L2 | 1030.47 | 3633.79 | 3.526342 |
| ITGB4BP | 1838.272 | 12978.61 | 7.060223 |  | ITGB4BP | 14030.75 | 23443.43 | 1.670861 |
| MAF1 | 233.6944 | 726.2614 | 3.10774 |  | MAF1 | 1010.869 | 2571.033 | 2.543389 |
| MRPL43 | 341.1313 | 1648.715 | 4.83308 |  | MRPL43 | 738.54 | 1569.04 | 2.124516 |
| MRPL52 | 942.6568 | 2716.687 | 2.881947 |  | MRPL52 | 1501.003 | 2304.313 | 1.535182 |
| MT1X | 683.2532 | 17664.34 | 25.85329 |  | MT1X | 8575.07 | 15393.53 | 1.795149 |
| MTMR4 | 171.9632 | 2537.057 | 14.75349 |  | MTMR4 | 569.2852 | 1069.938 | 1.879441 |
| MUL1 | 1732.673 | 4163.179 | 2.402749 |  | MUL1 | 5300.923 | 7994.674 | 1.508166 |
| MVP | 189.1773 | 1302.057 | 6.882734 |  | MVP | 3256.972 | 6634.201 | 2.036923 |
| MX1 | 267.426 | 569.575 | 2.129842 |  | MX1 | 389.7652 | 1488.296 | 3.818443 |
| SPP1 | 2137.452 | 20662.15 | 9.66672 |  | SPP1 | 8334.406 | 19483.71 | 2.337744 |
| STARD10 | 1001.348 | 1722.307 | 1.719988 |  | STARD10 | 392.2258 | 1375.038 | 3.505731 |
| STAT3 | 706.0753 | 1690.59 | 2.394348 |  | STAT3 | 1895.242 | 3163.69 | 1.66928 |
| SURF4 | 272.5316 | 9299.036 | 34.12095 |  | SURF4 | 4115.489 | 9471.796 | 2.3015 |
| TADA2B | 452.6806 | 1047.807 | 2.314672 |  | TADA2B | 445.6438 | 987.5822 | 2.21608 |
